# Development of Recombinant Protein-Based Vaccine Against Classical Swine Fever Virus in Pigs Using Transgenic *Nicotiana benthamiana*

**DOI:** 10.3389/fpls.2019.00624

**Published:** 2019-05-16

**Authors:** Youngmin Park, Dong-Jun An, SeEun Choe, Yongjik Lee, Minhee Park, Soohong Park, Sungmin Gu, Kyungmin Min, Nam Hyung Kim, Sangmin Lee, Jong Kook Kim, Hye-Yeon Kim, Eun-Ju Sohn, Inhwan Hwang

**Affiliations:** ^1^BioApplications Inc., Pohang, South Korea; ^2^Virus Disease Division, Animal and Plant Quarantine Agency, Gimcheon, South Korea; ^3^Protein Structure Group, Korea Basic Science Institute, Ochang, South Korea; ^4^Center for Convergent Research of Emerging Virus Infection (CEVI), Korea Research Institute of Chemical Technology, Daejeon, South Korea; ^5^Division of Integrative Biosciences and Biotechnology, Pohang University of Science and Technology, Pohang, South Korea; ^6^Department of Life Sciences, Pohang University of Science and Technology, Pohang, South Korea

**Keywords:** *Nicotiana benthamiana*, classical swine fever (CSF), DIVA vaccines, molecular farming in plants, plant-made vaccines

## Abstract

Classical swine fever virus (CSFV) is highly contagious, and fatal to infected pigs. Vaccines against CSFV have been developed from attenuated or modified live viruses. These vaccines are effective for immunization of animals, but they are associated with problems such as the accidental spreading of viruses to animals in the field, and with barriers to trade following vaccination. Here, we report the generation of transgenic *Nicotiana benthamiana* plants for large-scale, cost-effective production of E2 fusion protein for use as a recombinant vaccine against CSFV in pigs. Transgenic *N. benthamiana* plants harboring an intergenic, single-copy insertion of a chimeric gene encoding E2 fusion protein had high levels of transgene expression. For large-scale production of E2 fusion protein from leaf tissues, we developed a protein-purification protocol consisting of cellulose-binding domain (CBD)–cellulose-based affinity purification and size-exclusion gel-filtration chromatography. E2 fusion proteins showed high immunogenicity in piglets and provided protection against CSFV challenge. The CBD in the E2 fusion protein was also highly immunogenic. These results suggest that plant-produced recombinant E2 fusion proteins can be developed into cost-effective vaccines against CSFV, with the CBD as a marker antigen to differentiate between vaccination and natural infection.

## Introduction

Classical swine fever (CSF) is one of the most important viral diseases of domestic and wild pigs (Edwards et al., 2000). CSF is highly contagious and produces high fever, depression, anorexia, gastrointestinal symptoms, and conjunctivitis, leading to considerable mortality and substantial economic losses (Laevens et al., 1999; Fritzemeier et al., 2000; Moennig et al., 2003). CSF is caused by classical swine fever virus (CSFV), which belongs to the genus *Pestivirus* within the family *Flaviviridae* (Moennig, 2000). CSFV is an RNA virus with a single-stranded, positive RNA genome of approximately 12.3 kb that encodes a polypeptide of 3,898 amino acid residues (Rümenapf et al., 1993). This polypeptide is processed to produce 12 proteins, comprising four structural proteins (C, Erns, E1, and E2) and eight non-structural proteins (N^pro^, p7, NS2, NS3, NS4A, NS4B, NS5A, and NS5B) (Meyers et al., 1996). In addition, the genome contains a 5′ untranslated region (UTR) and a 3′UTR.

CSF is widely prevalent in pig populations in Europe, Asia, and South America (Greiser-Wilke et al., 2000; Tu et al., 2001; Deng et al., 2005; Cha et al., 2007; Postel et al., 2013). Moreover, the disease is endemic in some countries, including India, China, and Korea (Cha et al., 2007; Luo et al., 2014; Roychoudhury et al., 2014; Blome et al., 2017). Because of high mortality, outbreaks of CSF can cause significant damage to the swine industry (Saatkamp et al., 2000; Stegeman et al., 2000). CSF is therefore classified as a List A disease by the Oficina Internacional de Epizootias (OIE) (Edwards et al., 2000; OIE, 2013). To control the disease, vaccines derived from live, attenuated virus or the E2 subunit are commercially available (Paton et al., 2000; Deng et al., 2005; Blome et al., 2006; Suradhat et al., 2007). Vaccination with live, attenuated virus has been used in eradication programs throughout the world, and is still being used in endemically affected countries. However, in CSFV-free countries, prophylactic vaccination is usually prohibited. In this context, marker vaccines that can differentiate vaccinated animals from naturally infected animals are preferred, as they can reduce adverse impacts on trade (Blome et al., 2013).

There have been various attempts to develop marker vaccines for CSFV (Weiland et al., 1990; König et al., 1995). E2 protein, one of the coat proteins of CSFV, was identified as an antigen that can provide protective immunity, and has been used for the development of subunit vaccines (Depner et al., 2001; Uttenthal et al., 2001). The subunit vaccines can be safer than vaccines produced from attenuated live virus or modified live virus (Dong and Chen, 2007). E2 proteins produced in insect cells have been used as vaccines for pigs (Galliher-Beckley et al., 2015; Madera et al., 2016). In addition, E2 proteins produced in *Arabidopsis thaliana* are recognized by antibodies raised against native E2 proteins, and elicit production in mice of antibodies that recognize native E2 proteins (Sohn et al., 2018), suggesting that plant-produced proteins can be used as vaccines in target animals. Moreover, E2 proteins expressed transiently in *Nicotiana benthamiana* following transformation with *Agrobacterium tumefaciens* elicit production of CSFV-neutralizing antibodies in pigs and give protection from challenge with CSFV (Laughlin et al., 2018).

In this study, we investigated whether recombinant E2 fusion protein could be expressed at high levels in transgenic *N. benthamiana* plants by intergenic insertion of a single-copy transgene, and whether expressed fusion protein could be purified in a cost-effective manner for commercialization. Our results indicate that the E2 fusion protein is strongly immunogenic, protecting pigs against CSFV challenge. The recombinant E2 fusion protein was expressed at a high level, and purified at a low cost. Furthermore, the cellulose-binding domain (CBD) region of the fusion protein is highly immunogenic and can be used as a marker for animals vaccinated with the recombinant E2 fusion protein.

## Results

### Construction of an E2 Expression Vector and Protein Expression in Leaves of Transgenic *N. benthamiana*

**FIGURE 1:**
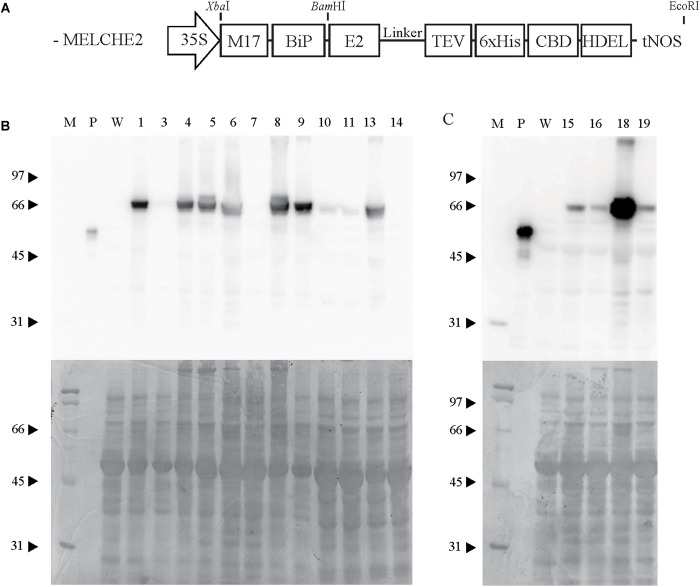
Design of the *MELCHE2* construct and selection of transgenic plants expressing recombinant E2 fusion protein at high levels. **(A)** Schematic view of the *MELCHE2* construct. 35S, double cauliflower mosaic virus 35S promoter; M17, 5′ untranslated region; BiP, endoplasmic reticulum (ER)-targeting signal peptide; E2, classical swine fever virus envelope glycoprotein E2; TEV, tobacco etch virus protease site; CBD, cellulose-binding domain of xynA; HDEL, ER-retention signal; tNOS, nos terminator. **(B,C)** Western-blot analysis of recombinant E2 fusion proteins expressed in T0 transgenic plants. Total protein was prepared from 16 independent lines at T0, and 50 μg of each extract was subjected to western blotting using anti-CBD antibody. Blots were stained with Coomassie Brilliant Blue after immunoblotting, as shown in the lower panel. M, protein standards (molecular weights in kD shown to the left); P, CBD positive control (GFP:CBD 10 ng); W, wild-type *Nicotiana benthamiana* used as a negative control.

To test the potential of recombinant E2 fusion protein for use as a vaccine, preparation of a large amount of the protein was necessary. *N. benthamiana* was assessed for protein production, because transgenic *A. thaliana* plants had previously been found to have a limitation for production of biomass (Sohn et al., 2018). Notably, *N. benthamiana* has been used as a host for production of many different recombinant proteins (Holtz et al., 2015). We previously demonstrated that a fusion construct containing the *E2* gene of CSFV results in a high level of protein expression in the endoplasmic reticulum (ER) of transgenic *A. thaliana* (Sohn et al., 2018). Accordingly, in this study, a similar construct with only minor differences was used (Figure 1A). The chimeric construct included BiP signal peptide and HDEL ER-retention signal, for accumulation of the fusion protein in the ER. A CBD was included as an affinity tag to facilitate purification. The tobacco etch virus (TEV) protease site was inserted between the E2 and CBD sequences, to enable removal of the CBD. The construct therefore directs expression of a BiP:E2:TEV:CBD:HDEL fusion protein. A 17 bp 5′ UTR (M17) that has demonstrated high translational efficiency (Kim et al., 2014) was also included. The final *M17:BiP:E2:TEV:CBD:HDEL* construct was named *MELCHE2*. The cauliflower mosaic virus (CaMV) 35S promoter and *A. tumefaciens* nopaline synthase gene (nos) terminator were used for expression of the chimeric construct.

The *MELCHE2* construct was inserted into the pCAMBIA1300 binary vector to give 1300::MELCHE2, which was introduced into *A. tumefaciens* and used for generation of transgenic *N. benthamiana* plants. Many putative transgenic plants (T0 plants) were obtained and examined for expression of plant-produced E2 fusion protein (ppE2) by western-blot analysis using anti-CBD antibody (Figure 1B,C). Extracts from most of these T0 plants produced a band at 66 kD, in contrast to the expected 58 kD of the E2 fusion protein (39 kD of E2 and 19 kD of other domains including CBD). One possible explanation for this discrepancy is that the ppE2 was N-glycosylated in *N. benthamiana*, as has been observed in *A. thaliana*. Notably, E2 protein has multiple N-glycosylation sites (Risatti et al., 2007). According to a glycosylation analysis algorithm^1^, E2 protein contains six putative N-linked glycosylation sites. The expression level of ppE2 varied considerably among the 16 transgenic plants that were screened, with transgenic line number 18 showing the highest expression. This line was therefore used in further experiments.

### Molecular Characterization of Transgenic Plants Expressing ppE2

**FIGURE 2:**
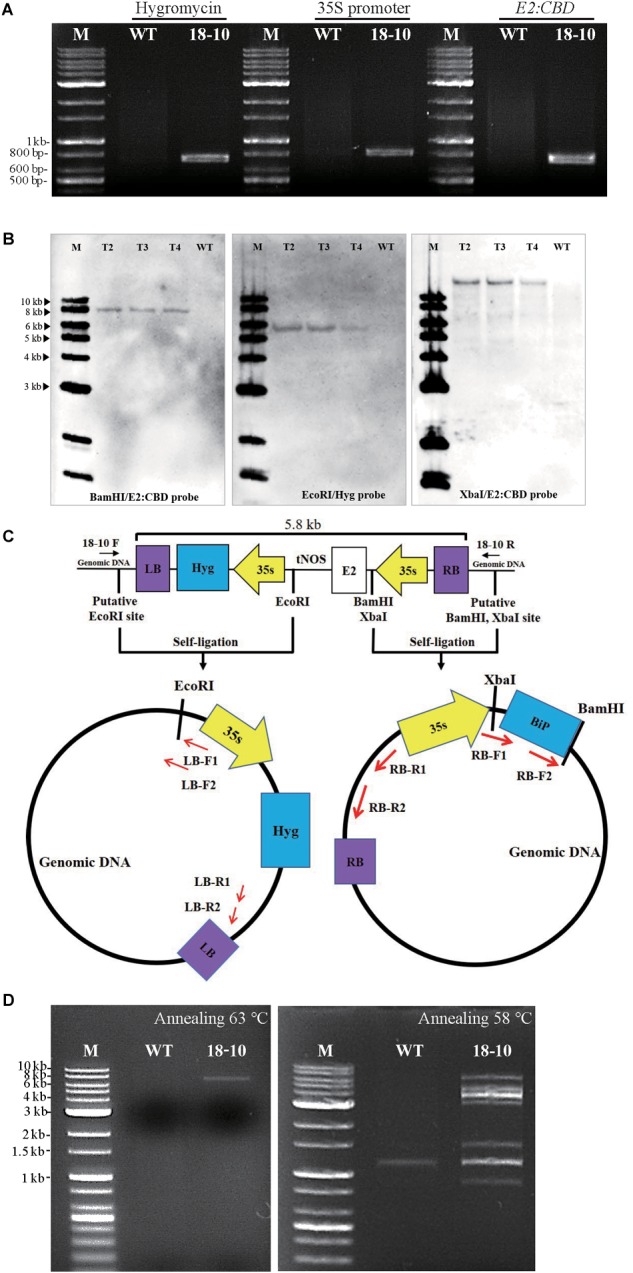
Transgenic plant line 18–10 contains only a single-copy insertion of the *MELCHE2* construct in an intergenic region. **(A)** PCR analysis of the T-DNA in transgenic *Nicotiana benthamiana* line 18–10. Genomic DNA was extracted and used for PCR amplification of hygromycin resistance gene, 35S promoter, and *E2:TEV:CBD:HDEL*. PCR products were analyzed by agarose-gel electrophoresis. M, DNA size markers (relevant sizes indicated to the left of the panel); WT, wild type. **(B)** Southern-blot analysis of transgenic line 18–10. Genomic DNA from T2, T3, and T4 generations of line 18–10 was digested with restriction endonuclease *Bam*HI, *Eco*RI, or *Xba*I, and probed with *E2:TEV:CBD:HDEL*-specific or hygromycin resistance gene-specific probes (hygromycin resistance gene). M, DNA size markers (relevant sizes indicated to the left of the panel); WT, wild type. **(C)** Schematic view of the fusion construct T-DNA, restriction sites of *Bam*HI, *Xba*I and *Eco*RI and primer sets used for iPCR. XbaI, upstream of BiP and BamHI, downstream of BiP were used for finding out RB insertion site. EcoRI, downstream of tNOS was used for LB insertion site. **(D)** Isolation of the full-length T-DNA in transgenic line 18–10 by PCR. The primer set was designed from the genomic DNA regions around the LB and RB insertion sites, as indicated in **(C)**. PCR was performed with genomic DNA of wild-type plants and transgenic line 18–10 as template. Two independent reactions were carried out at different annealing temperature 63^o^C (left panel) or 58^o^C (right panel), respectively. PCR products were analyzed by agarose-gel electrophoresis. M, DNA size markers (relevant sizes indicated to the left of the panel); WT, wild type.

We characterized transgenic line number 18. According to the guidelines for biosafety of genetically modified plants in South Korea^2^, a single copy of the transgene should be inserted in an intergenic region. We tested the copy number of the T-DNA by assessing hygromycin resistance. On hygromycin plates, T2 plants segregated with a 1:3 ratio of sensitive to resistant plants, indicating that transgenic line 18 has a single insertion of the T-DNA. We also determined the exact copy number of the transgene in line 18–10, one of the T3 homozygotic lines derived from transgenic line 18. First, we used PCR to detect the hygromycin antibiotic-resistance gene that is used for selection of transgenic plants, as well as the CaMV 35S promoter and E2:TEV:CBD regions of the *MELCHE2* construct. Total genomic DNAs from line 18–10 and from wild-type plants were prepared and used as templates for gene-specific PCR. The products were analyzed by gel electrophoresis. PCR with template DNA from transgenic line 18–10 produced specific bands for each of these targets, whereas template DNA from wild-type plants did not yield any bands (Figure 2A), indicating that transgenic line 18–10 contains the transgene as well as the drug-resistance gene. To determine the copy number, we performed Southern-blot analysis using genomic DNA from T2, T3, and T4 plants of transgenic line 18–10 and from wild-type plants. This DNA was digested with restriction endonuclease *Bam*H1, *Eco*R1, or *Xba*I, and separated on agarose gels. DNA digested with *Bam*H1 or *Xba*I was probed with a DNA fragment from the *E2:TEV:CBD* region, and DNA digested with *Eco*R1 was probed with a fragment from hygromycin resistance gene. For T2, T3, and T4 plants of transgenic line 18–10, each of these restriction-endonuclease treatments produced only a single band (Figure 2B), indicating that the transgene and hygromycin resistance gene are present as single copies in the host genome. No bands were observed following digestion of genomic DNA from wild-type plants.

To determine the exact point of insertion of the *MELCHE2* construct, we isolated the ends of the construct together with the neighboring genomic DNA by inverse PCR (iPCR) (Figure 2C). Genomic DNA was fragmented by treatment with *Bam*H1, which has a restriction site near to the BiP signal peptide, or with *Eco*R1, which has a site near to the nos terminator. DNA fragments produced by restriction digestion were self-ligated to produce circular DNA molecules, which were used as templates for iPCR. Amplified products between 0.8 and 1.5 kb were isolated, inserted into T-vector (Solgent; Cat No. SOT02-K020), and sequenced. The sequences were compared with the full genome sequence from the Sol Genomics Network database^3^, which showed that the genomic regions adjacent to right border (RB) and left border (LB) matched sequences in the *N. benthamiana* contigs Niben101Scf08318Ctg002 and Niben101Scf08318Ctg003, respectively (Supplementary Figure S1). A further sequence-similarity search was performed with the Basic Local Alignment Search Tool (BLAST)^4^, and the genomic DNA sequence around *MELCHE2* corresponded to the non-coding region upstream of the WAT1-related gene in *Nicotiana tomentosiformis*, indicating that the *MELCHE2* construct is inserted in an intergenic region. To further confirm this, we designed primers flanking the predicted insertion site and amplified the genomic DNA by PCR. When annealing temperature was 63°C, a DNA fragment containing T-DNA and genomic DNA region of approximately 7 kb was produced from the transgenic plants harboring the *MELCHE2* construct, but no bands were produced by PCR with template DNA from wild-type plants at an expected size (Figure 2D). As annealing temperature was lowered, the band composed of only genomic DNA region was observed from wild-type plants although multiple bands including a specific target band were detected in the transgenic plants. Taken together, these results suggest that a single copy of the fusion construct is inserted into an intergenic region.

### Purification and N-Terminal Sequencing of ppE2

**FIGURE 3:**
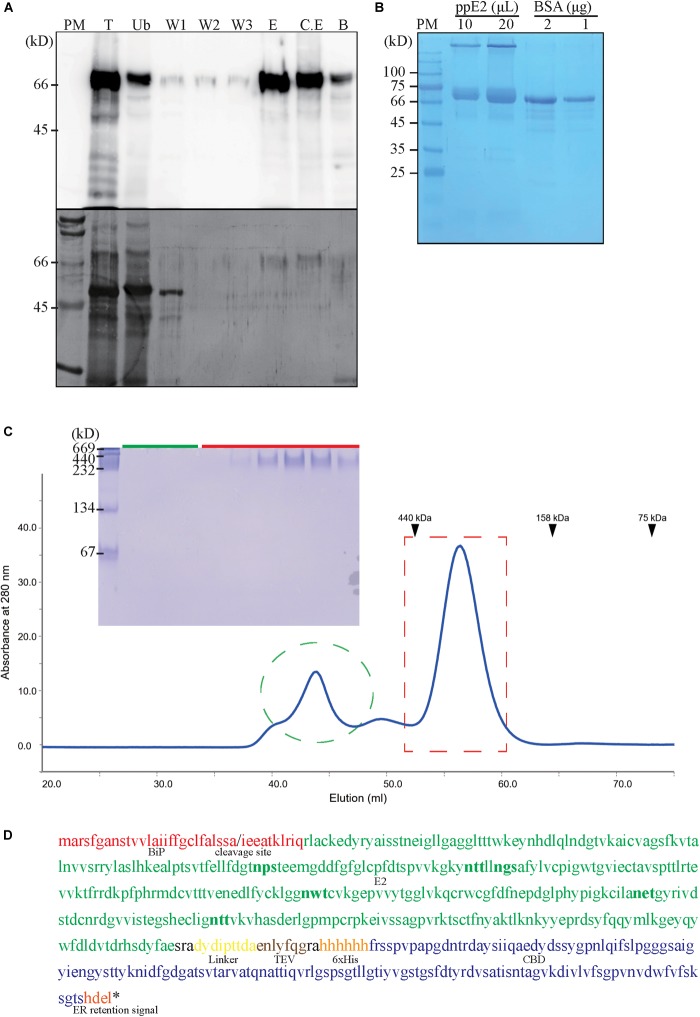
Purification and identity confirmation of recombinant E2 fusion protein. **(A)** Purification of plant-produced E2 fusion protein (ppE2). Protein extracts prepared from fresh leaves of transgenic *Nicotiana benthamiana* (1 g leaves/5 ml extraction buffer) were loaded onto a column containing 0.2 g of cellulose beads. The column was washed three times, and wash-off fractions were collected. Proteins were eluted with 2 × 5 ml of elution buffer. Aliquots of total protein (T), unbound fraction (Ub), wash-off fractions (W1–W3), and elution fractions (E1 and E2), as well as the post-elution cellulose-bead contents, were analyzed by western blotting using anti-CBD antibody. **(B)** Quantification of purified ppE2. Purified ppE2 was concentrated using 50 kD-cutoff centrifugal concentrators and analyzed by SDS-PAGE. Proteins were visualized by Coomassie Brilliant Blue staining. Bovine serum albumin (BSA) was used for comparison. **(C)** Size-exclusion chromatography and native-PAGE analysis of ppE2. Purified ppE2 was injected onto the equilibrated column and separated, with collection of 1.5 ml fractions. Fractions corresponding to absorbance peaks (marked with a green circle and a red box) were analyzed by 9% native PAGE and stained with Coomassie Brilliant Blue. Ferritin (440 kD), aldolase (158 kD), and conalbumin (75 kD) were used as molecular-weight standards. **(D)** Amino acid sequencing of ppE2 fusion protein. ppE2 (0.4 mg/ml) in PBS buffer containing cellobiose was subjected to Edman degradation in an automated manner. Amino acid residues in red indicate BiP residues of ppE2 following cleavage during endoplasmic reticulum targeting. Putative N-linked glycosylation sites are shown in bold.

To obtain plant-produced CSFV E2 antigen, extracts were prepared from 1 g of T3 plants of transgenic line 18–10 and used for purification of ppE2 by our previously established CBD-based affinity method (Sohn et al., 2018), with analysis of fractions by western blotting with anti-CBD antibody (Figure 3A). Following the application of total protein extract to the amorphous cellulose beads, the flow-through contained around 30% of unbound ppE2, compared to that of total extracts. The three wash fractions produced only faint bands at 66 kD on western blots, indicating that ppE2 binds tightly to the cellulose beads. The two elution fractions obtained by application of 1% cellobiose produced intense bands at 66 kD. After elution, little further protein was released by boiling the cellulose beads. Electrophoresis and protein staining demonstrated that the majority of cellular proteins were present in the unbound flow-through fraction (Figure 3A). A strong band at 55 kD, indicative of the large subunit of the rubisco complex, was observed in the first wash fraction, whereas no clear protein bands were observed in the other two wash fractions. The only visible protein bands in the two elution fractions corresponded to ppE2, indicating that it can be purified to near homogeneity by CBD-based affinity purification, as shown previously for extracts of transgenic *A. thaliana* harboring a similar construct to *MELCHE2* (Sohn et al., 2018).

Eluted protein fractions were concentrated 10-fold by centrifugal filtration, and aliquots were run alongside known amounts of bovine serum albumin (Figure 3B). By comparison of the intensities of protein staining, we estimated that the ppE2 concentration was 228 μg/ml, and that 16 μg of ppE2 could be obtained from 1 g fresh weight of plant leaves.

Electrophoresis of denatured purified ppE2 demonstrated the presence of a major band at 66 kD and a minor band at >180 kD, which suggested that ppE2 could form a high-molecular-weight complex. Notably, E2 proteins have previously been shown to form dimers (Lin et al., 2009; Hua et al., 2014). With size-exclusion gel-filtration chromatography, we found that purified ppE2 produced two peaks, at 45 and 55 ml elution volumes (Figure 3C). The 45 ml peak eluted earlier than a 440 kD marker protein. In non-denaturing electrophoresis, proteins from the 45 ml peak did not produce a band on the gel. These proteins may be aggregated forms of ppE2 that are too large to enter the gel. By contrast, the proteins from the 55 ml peak produced a band at ∼230 kD, suggesting that these proteins are multimers of ppE2. The occurrence of multimers larger than dimers might result from the presence of the additional domains in the E2 fusion protein.

Next, we examined the N-terminus of ppE2. The *MELCHE2* construct encoded a BiP signal peptide that should be removed after targeting to the ER, but the exact cleavage site is not known. We determined the N-terminal amino acid residue of the purified ppE2 by Edman degradation in an automated manner. Amino acid sequence analysis showed that the BiP signal peptide was cleaved between Ala27 and Ile28, resulting in 10 amino acids overhang at the N-terminus of ppE2 fusion protein (Figure 3D).

### ppE2 Generated in *N. benthamiana* Induces Production of CSFV-Recognizing Antibody in Piglets

**FIGURE 4:**
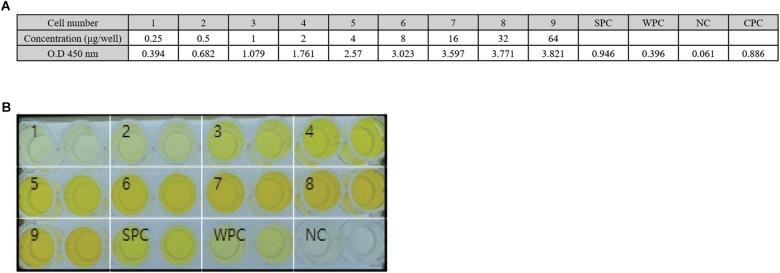
E2 fusion protein produced in *Nicotiana benthamiana* is recognized by an antibody to E2 of classical swine fever virus. **(A)** Quantification of the reactivity of plant-produced E2 fusion protein (ppE2) with anti-E2 antibody. Purified ppE2 was serially diluted and analyzed by ELISA, along with a strong protein control (SPC) and a weak protein control (WPC), with measurement of signal intensity at A_450_. **(B)** Visualization of the reactivity of ppE2 with anti-CSFV E2 antibody. Purified ppE2 was added to wells of an anti-CSFV E2 antibody-coated plate, and the reactivity was visualized using 3,3′,5,5′-Tetramethylbenzidine as substrate. Lanes 1–9, purified ppE2; SPC, strong positive control; WPC, weak positive control; NC negative control.

We assessed recognition of recombinant E2 fusion protein generated in *N. benthamiana* (0.25–64 ng/μl) by a commercially available E2-directed anti-CSFV ELISA (Figure 4). At 0.25 ng/μl, the recombinant protein signal was similar to that of the antigen provided as a weak positive control in the ELISA kit. The signal intensity increased with increasing concentrations of recombinant protein, and at >1 ng/μl it was stronger than that of the strong positive control.

**FIGURE 5:**
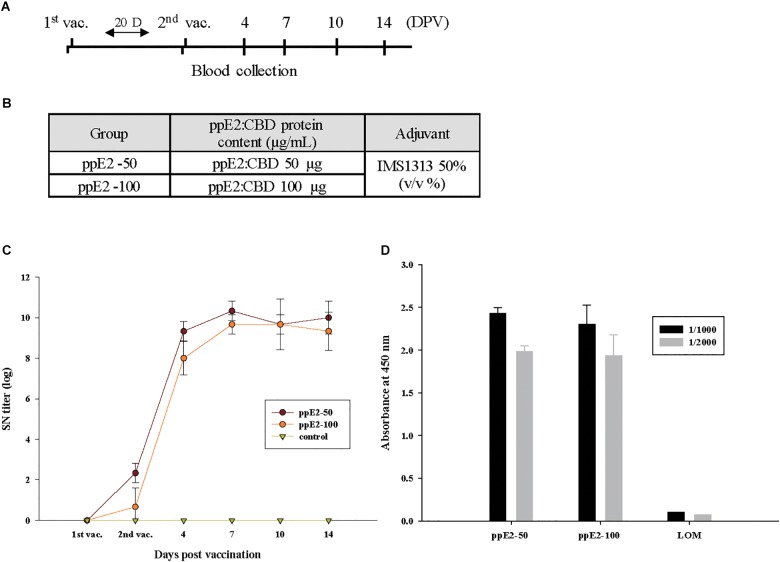
ppE2 is highly immunogenic and induces production of antibody against E2 in piglets. **(A)** Schematic presentation of the two-dose immunization protocol. DPV, days post-vaccination. Blood was collected immediately before injection and at the indicated DPV. **(B)** Immunization of piglets. The piglets were injected twice with 50 μg or 100 μg of plant-produced E2 fusion protein (ppE2) in 50% IMS1313 used as an adjuvant. For each dose, three piglets were immunized. **(C)** E2-specific immune response, determined by ELISA. Serum samples collected from piglets vaccinated with ppE2 were analyzed for E2-specific immune responses by ELISA. Non-vaccinated piglets were included as negative control. Data are mean ± SEM for three pigs per group. **(D)** Cellulose-binding domain (CBD)-specific immune response, determined by ELISA. Serum samples collected from piglets vaccinated with ppE2 or LOM live vaccine were diluted by 1:1,000 or 1:2,000 and used to analyze CBD-specific immune responses by ELISA. Data are mean ± SEM for three pigs per group.

We examined whether ppE2 generated in *N. benthamiana* could induce production of anti-E2 antibody in pigs. E2 fusion protein (50 or 100 μg) was mixed with the adjuvant IMS1313 and subcutaneously injected into piglets (Figure 5A,B). Each group included two animals. Before immunization, preimmune control serum was obtained from each animal. Neither experimental group had detectable levels of CSFV-recognizing antibodies at the time of the first injection, but these antibodies were detectable at the time of the second injection, and titers increased considerably thereafter, reaching maximal levels 7 days after the second injection (Figure 5C). No CSFV-recognizing antibodies were detected in control animals that were not immunized.

With the live CSFV vaccine that is currently used in Korea, vaccinated animals cannot be distinguished from naturally infected animals. We examined whether the presence of C-terminal CBD results in the production of anti-CBD antibodies that can function as markers for vaccination by ppE2 (Figure 5D). For an anti-CBD-specific ELISA, serum from immunized pigs was diluted 1,000- or 2,000-fold and incubated with 15 ng of CBD. No difference was observed between the ELISA signal intensities for serum samples from pigs vaccinated with 50 or 100 μg of ppE2. However, serum from animals vaccinated with the live CSFV strain LOM did not produce a signal in the anti-CBD ELISA. These results indicate that the CBD is an effective antigen that can be used as a marker to distinguish animals vaccinated with ppE2 from animals vaccinated with live virus or naturally infected in the field.

### ppE2 Produced in *N. benthamiana* Provides Protection Against CSFV in Piglets

**FIGURE 6:**
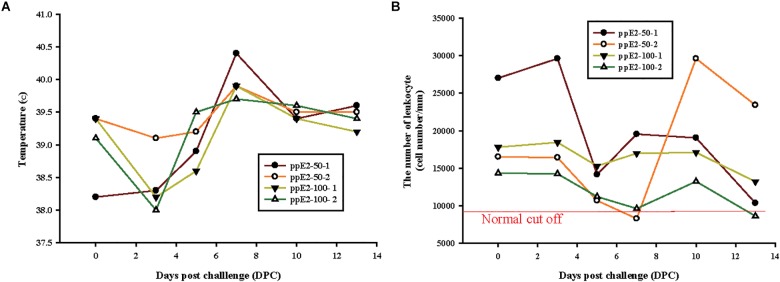
Plant-produced E2 fusion protein vaccination provides protection from classical swine fever virus challenge. **(A,B)** Two piglets were immunized with two doses of either 50 μg or 100 μg of plant-produced E2 fusion protein (ppE2), and challenged with virulent classical swine fever virus (CSFV). Symptoms of CSFV infection in vaccinated animals are shown. Body temperature **(A)** and total leukocyte counts **(B)** were examined at 0, 3, 5, 7, 10, and 13 days after viral challenge. The individual data points were presented.

To test whether ppE2 can provide protection against CSFV in target animals, we performed viral-challenge experiments. Piglets vaccinated with two doses of 50 or 100 μg of ppE2 were challenged with a virulent strain of CSFV (YC11WB) 2 weeks after the second vaccination, and clinical symptoms such as high fever and reduction in white blood cells (WBCs) were monitored after viral challenge. Regardless of the vaccination dose, all vaccinated piglets showed a normal range of body temperature (38–40°C) during monitoring for 13 days post-challenge (DPC) (Figure 6A). When WBCs were counted, Numbers of WBCs fluctuated, but remained above 9,000 cells per mm^3^, which is considered the lower limit of normal WBC levels (Figure 6B).

To assess viremia following the viral challenge, total RNA was prepared from the serum of vaccinated pigs at different time points and subjected to one-step RT-PCR using CSFV-specific primers to detect the 5′ non-coding region (5′ NCR) of CSFV (Table 1). In pigs vaccinated with 50 μg of ppE2, a positive reaction was observed only on day 7 post-challenge. No positive reactions were observed with a vaccination dose of 100 μg of ppE2. This result suggests that a higher vaccination dose might be more effective for protection of pigs from CSFV.

The presence of CSFV in the tissues of vaccinated animals following viral challenge was also assessed (Table 2). Total RNA was prepared from the tissues and analyzed by one-step RT-PCR using CSFV-specific primers. Tonsils from all piglets showed a positive reaction, indicating the presence of CSFV, whereas no CSFV RNA was detected in the other tissues.

### Single-Dose Vaccination With ppE2 Effectively Protects Pigs Against Virulent CSFV

**FIGURE 7:**
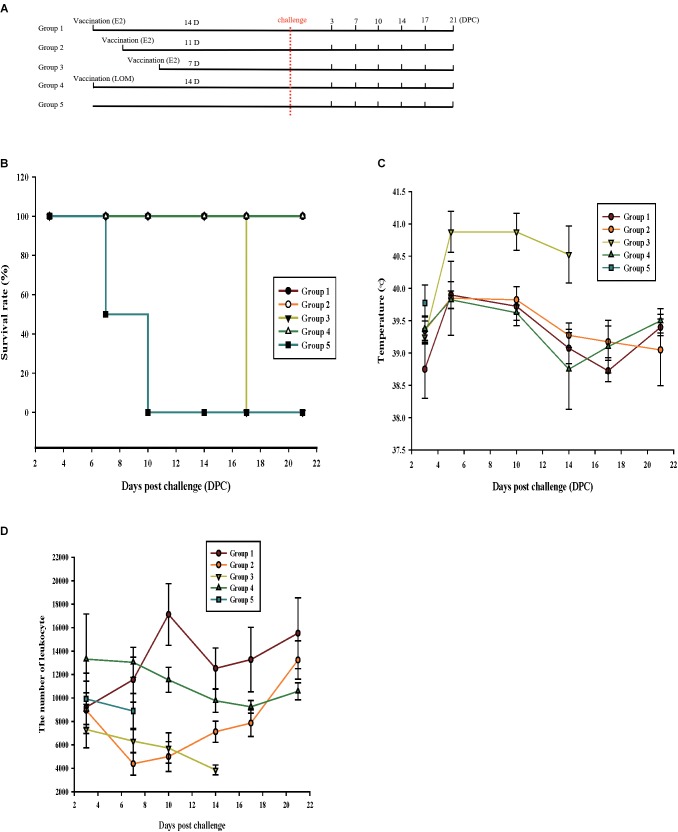
Single-dose vaccination with plant-produced E2 fusion protein can provide effective protection after 11 days. **(A)** Schedules of single-dose vaccination and challenge with virulent classical swine fever virus (CSFV). Piglets were vaccinated with plant-produced E2 fusion protein (ppE2) or live CSFV vaccine strain LOM. Survival and clinical conditions of vaccinated pigs after viral challenge were monitored at 0, 3, 7, 10, 14, 17, and 21 days post-challenge. **(B)** Survival of immunized and control (non-immunized) piglets. **(C,D)** Symptoms of CSFV infection in animals with single-dose vaccination. Body temperatures **(C)** and the number of WBCs **(D)** from all piglets were examined at the indicated time points until the animals died or were euthanized. Piglets from group 3 displayed abnormal clinical signs before death, and non-vaccinated pigs all died soon after challenge. Data are mean ± SEM for four piglets per group.

When animals are vaccinated, it is desirable to reduce the number of required injections. We examined whether single-dose vaccination with ppE2 can give long-lasting protection. Animals were injected once with ppE2 (100 μg) and then challenged with YC11WB strain. For a positive control, animals were vaccinated once with the LOM CSFV strain. Viral challenge was carried out at different times after vaccination of 10 weeks old piglets (four per group) (Figure 7A). All piglets were monitored for 21 days unless they were euthanized. Unvaccinated piglets and piglets with viral challenge 7 days post-vaccination all died after displaying symptoms such as leucopenia and high fever (Figure 7B–D). By contrast, piglets vaccinated with ppE2 and challenged 11 or 14 days post-vaccination, and piglets vaccinated with LOM and challenged 14 days post-vaccination, survived the CSFV challenge, although several showed temporary leucopenia and high fever. CSFV-neutralizing antibody titers were highest in piglets with viral challenge 14 days after ppE2 vaccination (Table 3), and titers in piglets with viral challenge 11 days after ppE2 vaccination were similar to those in piglets with viral challenge 14 days after LOM vaccination, indicating that ppE2 can provide full protection to animals from 11 days post-vaccination. These results suggest that single-dose vaccination with ppE2 effectively protects pigs against virulent CSFV challenge from 11 days post-vaccination, and maintains high levels of antibody titers up to at least 21 DPC.

## Discussion

To date, modified live vaccine has been used for protection of pigs against CSFV infection (Van Oirschot, 2003; Blome et al., 2006). However, modified live viruses are immunologically indistinguishable from wild-type CSFV in the field, highlighting the importance of developing vaccines for differentiation of infected from vaccinated animals. Plants are good bioreactors for vaccine production, because they produce clinical-grade materials quickly and are easily scalable for large-scale production (Holtz et al., 2015; Nandi et al., 2016). Therefore, we generated a recombinant construct containing *E2* of CSFV, expressed the recombinant E2 fusion protein in transgenic plants, and examined whether ppE2 could provide protection against virulent CSFV.

In general, transient expression has been shown to give higher levels of protein expression in plants than the transgenic approach (Yamamoto et al., 2018). Therefore, transient expression is typically favored when aiming for high yield in relation to biomass. Transient expression also does not require the lengthy screening process that is necessary to obtain a transgenic plant expressing the target gene at high levels (Garabagi et al., 2012). However, the transgenic approach also has some advantages, including avoidance of concerns relating to endotoxins that occur with the use of *A. tumefaciens* for transformation. In addition, because upstream processes such as agroinfiltration involving additional equipment and materials can be eliminated once the homozygous transgenic lines were generated, the transgenic approach is less laborious and is more cost-effective for protein production (Garabagi et al., 2012). Another advantage of the transgenic approach is its scalability once the expression line has been established. In this study, we selected a transgenic line with high expression of the recombinant E2 fusion protein, and determined that this expression was directed by a single copy of *MELCHE2* inserted in an intergenic region.

**Table 1:** Detection of viremia in serum from vaccinated piglets challenged with classical swine fever virus.

Group		Viremia rates at the day post challenge					
		0 DPC	3 DPC	5 DPC	7 DPC	10 DPC	13 DPC
ppE2-50	1	–	–	–	**+**	–	–
	2	–	–	–	**+**	–	–
No. of (+) reaction/ Total		0/2	0/2	0/2	2/2	0/2	0/2
ppE2-100	1	–	–	–	–	–	–
	2	–	–	–	–	–	–
No. of (+) reaction/ Total		0/2	0/2	0/2	0/2	0/2	0/2

*(−); undetectable; viremia rates at each day post-challenge (DPC) was detected by RT-PCR as described in Materials and Methods section.*

**Table 2:** Detection of CSFV RNA in several tissues after challenge.

Group		The presence of CSFV at the animal tissues									
		Tonsils	Heart	Liver	Lung	Kidney	Spleen	Mesenterv	Ileum	Caecum	Brain
ppE2-50	1	+	–	–	–	–	–	–	–	–	–
	2	+	–	–	–	–	–	–	–	–	–
No. of (+) reaction /Total		0/2	0/2	0/2	0/2	0/2	0/2	0/2	0/2	0/2	0/2
ppE2-100	1	+	–	–	–	–	–	–	–	–	–
	2	+	–	–	–	–	–	–	–	–	–
No. of (+) reaction /Total		0/2	0/2	0/2	0/2	0/2	0/2	0/2	0/2	0/2	0’2

*(−); undetectable; RNA was extracted from each tissue, and CSFV was detected by RT-PCR as described in Materials and Methods section.*

For economical large-scale production of recombinant proteins, it is essential that the costs associated with protein purification should be minimized. Our strategy was to use CBD for the purification of the fusion protein. Cellulose is an inexpensive and readily available biomaterial that can be used as a matrix for affinity-based protein purification, and the CBD has high affinity to amorphous cellulose (Wan et al., 2011; Sugimoto et al., 2012; Armenta et al., 2017). Thus, CBD–cellulose binding is the basis of a cost-effective method for protein purification. Previously, we successfully used this approach to purify a similar recombinant E2 fusion protein from *A. thaliana* (Sohn et al., 2018). Here, this method enabled purification of ppE2 from extracts of *N. benthamiana*. Although E2 proteins naturally exist as dimers, our results suggested that ppE2 occurred as a multimer.

In the development of vaccines from plant-produced recombinant proteins, the most important target variable is immunogenicity. The ppE2 generated from *N. benthamiana* was recognized in a commercially available ELISA by an antibody directed against CSFV E2 protein. In addition, a protocol of priming and boost injections of piglets with 50 or 100 μg of ppE2 produced anti-E2 antibodies and provided effective protection against virulent CSFV challenge. The vaccinated piglets did not display severe clinical symptoms such as viremia, high fever, and leucopenia, suggesting the possibility of using ppE2 as a vaccine against CSFV. However, there was no clear correlation between the amount of E2 injected into animals and antibody titers or a few symptoms after virus challenge. One possible explanation is that this may be due to the condition of each pig. In the viral challenge experiments, the number of pigs in each group was only two, thereby leading to high data variations. Thus, the data were not suitable for statistical analysis. Thus, these results require cautious interpretation and experiments should be repeated with a sufficient number of piglets in the future study. Nevertheless, single-dose vaccination provided effective protection against CSFV challenge, which was comparable to that of the LOM vaccine that is currently used in the field in Korea, further supporting the potential of ppE2 as a subunit vaccine against CSFV in pigs. These results are consistent with previous findings that single-dose vaccinations with E2 produced in insect or plant expression systems effectively protect pigs against CSFV infection (Galliher-Beckley et al., 2015; Madera et al., 2016; Laughlin et al., 2018). Here, we found that a single-dose ppE2 vaccination with 100 μg requires 11 days to give full protection from CSFV infection, similar with commercial E2 subunit vaccines which give full protection at 14 days post-vaccination but partial protection before 10 days post-vaccination (Bouma et al., 2000; Uttenthal et al., 2001). This result proposes that not only MLV vaccine but also E2 subunit vaccine can be useful in an emergency situation. However, a new immunization protocol is needed for the early onset of protection. To obtain protection at earlier time points, one possible solution is to use greater amounts of ppE2 for vaccination. In fact, we used 100 μg of ppE2 for single-dose vaccination, whereas Laughlin et al. (2018) used 200 μg of E2. We also noticed the difference in the protein composition; ppE2 protein fused with CBD in this study existed as multimer whereas E2 protein of Laughlin et al. (2018) had the native structure, dimer, raising the possibility that the difference in the E2 protein composition may affect the antigenicity. The effectiveness of such a strategy could be tested in future research.

**Table 3:** Single-dose vaccination and classical swine fever virus (CSFV)-neutralizing antibody titers before and after viral challenge.

Group	Pig#	Before vac-cination	3 DPV	7 DPV	10 DPV	14 DPV	3 DPC	7 DPC	10 DPC	14 DPC	17 DPC	21 DPC
G1	51	<4	<4	<4	4	32	64	128	2048	2048	1024	4096
	52	8	8	8	8	32	32	256	2048	4096	2048	4096
	53	4	4	<4	16	64	256	1024	2048	4096	2048	2048
	54	<4	<4	<4	8	8	32	512	2048	2048	1024	2048
Group	Pig#		before vac-cination	4 DPV	7 DPV	11 DPV	3 DPC	7 DPC	10 DPC	14 DPC	17 DPC	21 DPC
G2	55		4	16	4	32	32	64	128	512	512	1024
	56		4	8	16	32	16	128	512	1024	2048	1024
	57		4	<4	8	4	4	64	512	256	1024	2048
	58		<4	8	16	16	16	64	1024	1024	1024	1024
Group	Pig#			before vac-cination	3 DPV	7 DPV	3 DPC	7 DPC	10 DPC	14 DPC	17 DPC	21 DPC
G3	59			<4	<4	<4	4	4	<4	8	16	NA
	60			<4	<4	<4	4	4	4	8	16	NA
	61			<4	<4	<4	8	16	32	32	32	NA
	62			<4	<4	<4	16	8	16	16	NA	NA
Group	Pig#	before vac-cination	3 DPV	7 DPV	10 DPV	14 DPV	3 DPC	7 DPC	10 DPC	14 DPC	17 DPC	21 DPC
G4	63	8	16	16	8	32	64	64	256	1024	1024	1024
	64	8	4	8	16	32	128	256	256	1024	1024	1024
	65	<4	<4	8	32	32	128	128	128	512	512	512
	66	4	<4	16	32	128	128	128	256	512	512	512
Group	Pig#	before vac-cination	3 DPV	7 DPV	10 DPV	14 DPV	3 DPC	7 DPC	10 DPC	14 DPC	17 DPC	21 DPC
G5		8	8	4	4	4	4	<4	NA	NA	NA	NA
		<4	<4	<4	<4	<4	<4	<4	NA	NA	NA	NA
		<4	<4	<4	<4	<4	<4	<4	NA	NA	NA	NA
		<4	<4	<4	<4	<4	<4	<4	NA	NA	NA	NA

*DPC, days post-challenge; DPV, day post-vaccination; NA, not available. CSFV-neutralizing antibody titers were measured as described in the Materials and Methods section.*

## Conclusion

In conclusion, we successfully generated transgenic *N. benthamiana* plants with single-copy insertion of the *MELCHE2* construct in an intergenic region. These plants produced recombinant E2 fusion protein, enabling us to purify the protein from extracts of *N. benthamiana* leaf tissues to high purity. We demonstrated the antigenicity and immunogenicity of ppE2, which provided effective protection against CSFV infection following priming and boost injections of piglets. We also demonstrated the possibility of single-dose vaccination with 100 μg of ppE2, with the caveat that at least 11 days were required for the development of protection.

## Materials and Methods

### Plant Growth Conditions

*N. benthamiana* plants were grown on soil composed of zeolite (30 g), pearlite (130 g), coco-peat (705.3 g), peat moss (130 g) and wetting agent (0.1 g), and heat-treated at 80°C for 8 h before use. The culture room was maintained 25°C ± 2°C and 50% ± 5% relative humidity under a 16:8 h light:dark cycle. The light source was LED lamp and intensity was 150 μmol⋅m^−2^⋅s^−1^.

### Plasmid Construction and Generation of Transgenic Plants

We used transmembrane domain deleted E2 derived from LOM strain, which is commercially used in South Korea as a modified live vaccine. To generate the *MELCHE2* construct, a translation-enhancing sequence (M17; 5′-GGCGTGTGTGTGTGTTAAAGA-3′) was added as the 5′ UTR upstream of BiP signal peptide by use of the M17/BiP-F forward primer, which contains both the M17 sequence and 20 nucleotides (nt) of *BiP1* (nucleotide positions 1–20) (Kim et al., 2014). The reverse primer consisted of 6 nt of the *Bam*HI restriction site and 21 nt of *BiP1* (nucleotide positions 252–272) (Supplementary Table S1). The *E2* coding sequence was codon-optimized for expression in *N. benthamiana* (Bioneer corp., Daejeon, Korea) and amplified by PCR using primers E2-F and E2-R (Supplementary Table S1). E2-F contained 6 nt of the *Bam*HI restriction site and 22 nt of the *E2* coding sequence (nucleotide positions 1–22), and E2-R consisted of 6 nt of the *Xma*I restriction site and 21 nt of the *E2* coding sequence (nucleotide positions 1,002–1,023). The *TEV:CBD:HDEL* fragment was generated by PCR. The forward primer consisted of 29 nt of a linker sequence, 21 nt of the TEV protease site, and 20 nt of *Clostridium stercorarium xynA* (nucleotide positions 1,554–1,574), and the reverse primer contained 20 nt of *xynA* (nucleotide positions 1,954–1,974) and 12 nt of HDEL (His-Asp-Glu-Leu), which includes 6 nt of the *Sac*I restriction site. The resulting PCR products were digested with restriction endonucleases *Bam*HI and *Xma*I and ligated into, similarly, digested pCAMBIA1300 binary vector, to generate 1300MELCH. To generate transgenic plants expressing *MELCHE2* encoding the E2 fusion protein, 1300MELCH was introduced into *A. tumefaciens* strain LBA4404, following a previously described method (Horsch et al., 1985). *N. benthamiana* leaves were cut into 0.5 cm × 0.5 cm pieces and incubated in Murashige and Skoog (MS) medium containing 2.0 mg/l α-naphthaleneacetic acid (NAA), 0.5 mg/l 6-benzylaminopurin (6-BAP), and 100 μl of a culture of *A. tumefaciens* transformed with 1300MELCH, for 10 min. The solution was removed by blotting with sterilized filter paper. Leaf segments were placed upside down on MS-agar plates containing 1.0 mg/l NAA and 0.5 mg/l 6-BAP, and incubated for 3 days. They were then washed with 10 ml of MS containing 1.0 mg/l NAA and 0.5 mg/l 6-BAP, which was removed with sterilized filter paper. Leaf segments were then placed upside down on MS-agar plates containing 1.0 mg/l NAA, 0.5 mg/l 6-BAP, 200 mg/l kanamycin, and 250 mg/l cefotaxime, and incubated in the dark for 7–10 days. Leaf segments were then transferred to the light to induce shoot and root formation, on MS-agar plates containing 200 mg/l kanamycin and 250 mg/l cefotaxime. Transformed plants were transferred to soil, and expression of the E2 fusion protein was analyzed by western blotting. Seeds were harvested from transgenic plants with high expression, and homozygous plants were selected at T3 generation based on the 3:1 segregation ratio in the presence of antibiotics.

### Protein Purification and Western Blotting

Leaf tissues from transgenic plants were harvested and ground in liquid nitrogen. Total protein extracts were prepared in five volumes of protein-extraction buffer (50 mM NaOAc, pH 5.2, 50 mM NaCl, 1 mM CaCl_2_, 0.1% Triton X-100) and incubated on ice for 30 min. Homogenates were centrifuged at 20,000 × *g* for 30 min, and supernatants were collected. Total protein extract (5 ml) was loaded onto a column containing 0.2 g of amorphous cellulose beads (Sigma-Aldrich, CAS Number 9004-34-6) at a flow rate of 100 μl/min, and washed three times with 5 ml of washing buffer (50 mM NaOAc, pH 5.2, 50 mM NaCl, 1 mM CaCl_2_) at a flow rate of 500 μl/min. Recombinant E2 fusion protein was eluted from the cellulose matrix with 2 × 5 ml of elution buffer (50 mM Tris-HCl, pH 8.8, 50 mM NaCl, 1 mM CaCl_2_, 1% cellobiose). Total protein and the fractions from flow-through, washing-off, and elution, as well as the post-elution cellulose beads, were separated by 10% SDS-PAGE and analyzed by western blotting with anti-CBD antibody (In-house). The blots were finally stained with Coomassie Brilliant Blue R-250 (Biosolution, Suwon, South Korea, Cat. No: BC006).

### Size-Exclusion Chromatography and Native PAGE

For size-exclusion chromatography, the ÄKTA Prime chromatography system and a HiLoad^TM^ 16/60 Superdex 200 pg (GE Healthcare, Madison, WI, United States) column were used. The column was washed and equilibrated with two column volumes (120 ml) of 50 mM Tris-Cl, pH 7.4, 300 mM NaCl, and 0.5 mM EDTA prior to sample injection. A concentrated protein sample was centrifuged at 18,000 × *g* at 4°C for 20 min with a PURISPIN 17R microcentrifuge (CRYSTE Separation Technology, United Kingdom), and the soluble supernatant was carefully injected into the system with a 1 ml syringe. The chromatography was performed at 1 ml/min, and 1.5 ml fractions were collected, with monitoring of protein elution by absorbance at 280 nm. Fractions corresponding to absorbance peaks were analyzed by native PAGE on a 9% gel (5.15 ml of distilled water, 2.9 ml of 30% acrylamide/bis-acrylamide (37.5:1), 2 ml of 5 × buffer [14.64 g/l imidazole, 41.7 g/l HEPES], 50 μl of 10% ammonium persulfate, 10 μl of tetramethylethylenediamine). Ferritin (440 kD), aldolase (158 kD), and conalbumin (75 kD) (GE Healthcare) were used as protein molecular-weight standards. The gel was stained with Coomassie Brilliant Blue R-250.

### N-Terminal Amino Acid Sequencing

For N-terminal amino acid sequencing of recombinant E2 protein, purified protein was transferred from a SDS-PAGE gel to a polyvinylidene difluoride membrane, and the transferred protein was analyzed with a Protein Sequencing System (model 492cLC, Applied Biosystems, Foster City, CA, United States) at Korea Basic Science Institute.

### Antigenicity of ppE2

Purified ppE2 was serially diluted. Sandwich ELISA was performed with the VDPro CSFV AG ELISA kit (Median Diagnostics, Chuncheon, South Korea; Cat. No. ES-CSF-03), which contains a monoclonal antibody to CSFV E2 protein. An antibody-coated 96-well plate was incubated at room temperature for 30 min before use. Dilution solution and sample (50 μl each) were added to each microplate well. Positive and negative controls were diluted twofold, and 50 μl of each control was added to a microplate well. The plate was incubated at 37°C for 1 h, the reaction solution was removed, and the plate was washed three times with 300 μl per well of 1× wash buffer. Then, 100 μl of horseradish-peroxidase (HRP)-conjugated anti-CSFV antibody was added to each well, incubated at 37°C for 1 h, and washed three times with 300 μl of 1× wash buffer. 3,3′,5,5′-Tetramethylbenzidine (TMB) substrate (100 μl) was added to each well and incubated at room temperature for 10 min, then 50 μl of stop solution was added. Finally, the absorbance of the solution in each well was read at 405 nm on an ELISA Reader.

### Vaccination of Piglets and Challenge With CSFV

Piglets were randomly assigned to groups to receive intramuscular injections with either 50 or 100 μg of ppE2 combined with adjuvant IMS1313, or LOM vaccine. At 20 days after the first injection, a second injection was given with the same dose as the first. Animals in a control group were injected with phosphate-buffered saline (PBS). Serum was collected immediately prior to the first and second injections, and 4, 7, 10, and 14 days after the second injection, for examination of the levels of anti-E2 antibodies. The piglets were challenged with YC11WB, a virulent CSFV strain, at a virus concentration of 10,000 × LD_50_, via intramuscular and intranasal routes. Clinical symptoms were monitored during the study, and blood samples were collected at 0, 3, 5, 7, 10, and 13 DPC and used for leukocyte counting and antigen detection. Leucopenia was defined as <9,000 cells/mm^3^.

### ELISA Assay Against CBD

For antigen coating, 100 μl of CBD protein (0.15 ng/μl) was added to 96-well plates and incubated with CB buffer (15 mM Na_2_CO_3_, 35 mM NaHCO_3_ and 3 mM NaN_3_) at 4°C for overnight. After eliminating solution, the wells were washed with PBS-T buffer (PBS buffer + 0.1% Tween 20) for three times. Then, 100 μl of a blocking solution (3% skim milk and PBS-T buffer) was added and incubated at 37°C for 1 h. 1,000- or 2,000-fold diluted serum from piglets vaccinated with ppE2 or LOM was added to each microplate well and incubated at 37°C for 2 h. The wells were washed with PBS-T and D.W. for three times, respectively. HRP conjugated anti-pig antibody was diluted by 5,000 and 100 μl of the antibody was added to each well. After 1 h incubation at 37°C, the wells were washed with PBS-T and D.W. for three times, respectively. Hundred microliter of TMB substrate was added to each well and incubated at room temperature for 10 min. In order to stop the reaction, 50 μl of 2 M H_2_SO_4_ was added and the absorbance of the solution in each well was read at 405 nm on an ELISA Reader.

### Serological Examination

Aliquots (50 μl) of twofold serially diluted serum were mixed with 50 μl of 200 × [Median Tissue Culture Infectious Dose] (TCID_50_) of CSFV LOM strain in a 96-well plate, and incubated at 37°C for 1 h in the presence of 5% CO_2_. CPK-16 cells (100 μl of 2 × 10^5^ cells/ml) in Minimum Essential Medium (MEM) – α Modification (Gibco; Cat. No. 12571063) containing 10% fetal bovine serum (Biowest; Cat. No. S1520-500), 1× antibiotic–antimycotic (Gibco; Cat. No. 15240062), 1× sodium pyruvate (Gibco; Cat. No. 25030081), 1× MEM Non-Essential Amino Acids (Gibco; Cat. No. 11140050), and 1× L-glutamine (Gibco; Cat. No. 11360070) were added to all wells of the plate. At 72 h post-inoculation, the cells were fixed with 100 μl of pre-chilled 80% acetone for 7 min at −20°C. Neutralizing peroxidase-linked assay was performed with commercial anti-LOM antibody (Median Diagnostics; Cat. No. 9011) and biotinylated goat anti-mouse IgG antibody (Vector Lab; Cat. No. BA-9200). Anti-LOM antibody (100 μl of 200-fold dilution) was added and incubated for 1 h at 37°C. Wells were washed with PBS three times, and 100 μl of 200-fold diluted secondary antibody was added and incubated for 1 h at 37°C. After washing three times with PBS, VECTASTAIN ABC-HRP Kit (Vector Lab; Cat. No. PK-4000) was added according to the manufacturer’s instructions and incubated for 1 h at 37°C. After washing three times with PBS, ImmPACT DAB Peroxidase (HRP) Substrate (Vector Lab; Cat. No. SK-4100) was added according to the manufacturer’s instructions. CSFV-neutralizing antibody titers in serum samples were expressed as the reciprocal of the highest dilution that caused 50% neutralization.

### Detection of CSFV RNA in Blood and Tissues

To examine the presence of CSFV in infected animals, RNA was isolated from serum and from tissues (tonsils, heart, liver, lung, kidney, spleen, mesentery, ileum, caecum, and brain) with an RNeasy mini kit (Qiagen; Cat No. 74104). Total RNA was subjected to one-step RT-PCR using VDx CSFV 5′ NCR RT-PCR (Cat No. NS-CSF-11). Total RNA (5 μl) and CSFV primer mix (15 μl) were combined and incubated according to the manufacturer’s instructions. PCR products were separated on 1.5% agarose gels, and the presence of the 5′ NCR band was determined after staining with ethidium bromide.

### Single-Dose Vaccination and Challenge Experiments

Piglets were randomly assigned to five groups and vaccinated with 100 μg of ppE2 (groups 1–3) or LOM (group 4). Piglets in the control group (group 5) received PBS only. ppE2-vaccinated piglets in groups 1, 2, and 3 were kept for 14, 11, and 7 days, respectively, prior to virus challenge. Piglets vaccinated with LOM were kept for 14 days before challenge. All piglets were challenged with YC11WB, a virulent CSFV strain, at a virus concentration of 10,000 × LD_50_, via intramuscular and intranasal routes. Clinical symptoms such as fever and bodyweight were monitored during the study. Serum was harvested at 3, 7, 10, 14, 17, and 21 DPC and used for examination of the titers of neutralizing antibodies, and for WBC counts.

## Data Availability

All datasets for this study are included in the manuscript and the Supplementary Files.

## Ethics Statement

All animal experiments complied with the current laws of South Korea. Animal care and treatment were conducted with guidelines established by the Animal and Plant Quarantine Agency Animal Care and Use Committee (QIA-ACUC). The study was approved by QIA-ACUC with permit number 2017-369.

## Author Contributions

IH, E-JS, and YL designed and organized the overall study. YP and IH wrote the manuscript. D-JA, SC, and JK organized and carried out immunization-related work and antibody analysis. MP performed cloning and generated constructs. SP, SG, and KM carried out protein purification and western blot analysis. NK performed ELISA assay. H-YK provided information for N-terminal amino acid sequence of plant-produced E2. All the authors agreed on the contents of the manuscript and post no conflicting interest.

## Conflict of Interest Statement

The authors YP, MP, SP, SG, KM, NK, SL, JK, and E-JS were employed by company BioApplications Inc. The remaining authors declare that the research was conducted in the absence of any commercial or financial relationships that could be construed as a potential conflict of interest.

## Footnotes

1 http://www.cbs.dtu.dk/services/NetNGlyc/

2 http://www.nih.go.kr

3 https://solgenomics.net

4 https://blast.ncbi.nlm.nih.gov/Blast.cgi
