# Supplementary material for: Development of Recombinant Protein-Based Vaccine Against Classical Swine Fever Virus in Pigs Using Transgenic Nicotiana benthamiana
Source: Front Plant Sci. 2019 May 16;10:624. doi: 10.3389/fpls.2019.00624 (PMC6531818; doi:10.3389/fpls.2019.00624)
Supplement: Supplementary file 1 [file Image_1.pdf]

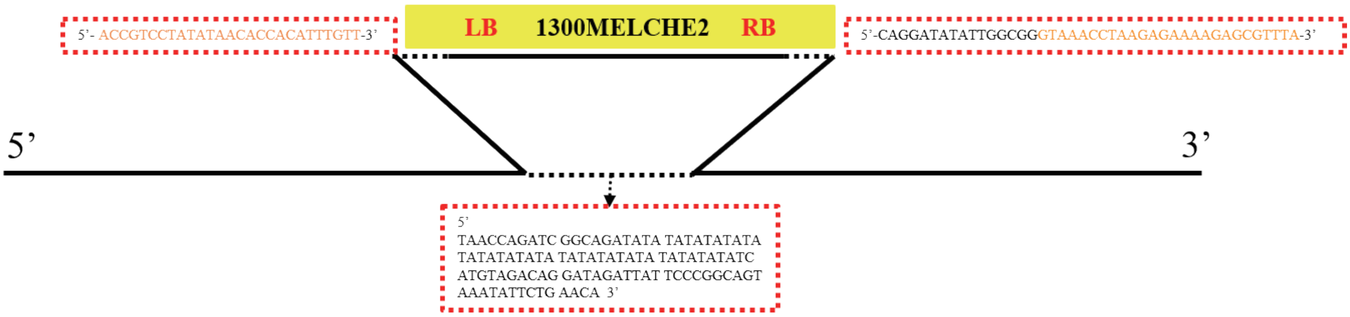

3'
ACGGGCCCGATACCAGTACGTTCTATTCTAATTTCATTTCCAATTTCCTTAAATATTTTCAGAAAATAACTTTACACAAAAATTTATTTCTCTGACTTTGAACCTC
GGAATTCAATTCCGGACATATGACCAAGTCACATATTTTCCTACGGACTCTTCGAGACCGTCAAATCACGGGTCTGAGTTCATTTATCCTAAAAGTTGACCAGA
GTCAAATTTATTCAACTTAATATCAAAATTTAGTATTTTTCTCACAGGATTCATATTTAAGCTTTCTGGCTACGCGCTGGACTGCATATGCAAATCGAGGCAACT
CCAGATGAGGTTTTAAGGCCTCAAAAGTACATAATATATAAAAAAATAAGTGATGACCCCTTCGAGTCGTCACAATAACCAACCTACGCATGTCTCTCATGTGTG
TATGTAATGACTAAAATATCCTCATTTAACTATCTTAACTCGTGAATAATTAATACACCTTATTCCGTATTATTCTTATACACTTACCAAACGACTCCTTCGCTAA
GAACAAAAAAAAGATACGGGGTAAGGAAGAAAAACCAATAACATTAGAAACCACCATATGCAAATTAAGCTGAGGTCCACAAAGGAATTGATTGCTTTTTTTAT
TTTTTTAATTCAAACCACRB **1300MELCHE2** LBATAATGTTGATTCTTGATTTTTCGTTTCTTTTTTTAATATTGATGAGCCCCGAGCAAGTTTCTAACATATAGT
ATATGAATAATATCATCAGTAACATACATAAAAGTTTAGACAAGTAGTGTAGTATTACTCTCTTTTTTTCTCTTTGTATATCGTTTCTTATTAATTAATAGGTCTT
AGAATAATGGTAAAATTATTTTCGTATGATTTATATGTTAGGCATTTGAACCTTATAAATAGTTATTAATATTTATATCAGAATAAATTAACTACATATCTTTAGA
GTACGGTTCATTCTCAAATCCCAGGTGAATACAAAATACTCTATATATCGGCTGCCTTTTTATATCGTTTCTTATTAGTAGTTAAGAAAGGATAGATGAAACCCT
GCACTGATACAAGTGCTGAGATATACGTACGTTGCTAGACAAAACTTTTGAGACAAGTTTAAGACTTTAAGGTAAAATTAAAATAGCTATACGTGGTGGAACAG
ATCCAAGAAGTCTACAGCTTAAACTAAACTCGTGCCCTGTTAGAATAAATATATCACATTGAAAGCAATCATACAATTTTATTCATGTGTAAATCAAACAATTAA
CACATGCAAAAATTATATGAGTTATCTTTTGAAC **5'**

Supplementary Figure S1. The nucleotide sequence of the insertion site of the MELCHE2 construct.

It was predicted that approximately 104 nucleotides were deleted by insertion of the construct and additional 16 nucleotides around right border (RB) were eliminated, as indicated by red boxes. While green letters display the nucleotide sequence of the *Nicotiana benthamiana* contig, Niben101Scf08318Ctg002, that matched with the sequence obtained from the RB iPCR product, blue letters show Niben101Scf08318Ctg003 that matched with the sequence obtained from the left border (LB) iPCR product.
