# Supplementary material for: Development of Recombinant Protein-Based Vaccine Against Classical Swine Fever Virus in Pigs Using Transgenic Nicotiana benthamiana
Source: Front Plant Sci. 2019 May 16;10:624. doi: 10.3389/fpls.2019.00624 (PMC6531818; doi:10.3389/fpls.2019.00624)
Supplement: Supplementary file 2 [file Table_1.pdf]

Supplementary Table S1. Nucleotide sequences of primers used in this study.

M17/BiP-F	GGCGTGTGTGTGTGTTAAAGAATGGCTCGCTCGTTTGGAGC
BiP-R	CGGGATCCTTAACTTCGTAGCCTCTTCT
E2-F	CGGGATCCAACGGCTAGCCTGCAAGGAAGA
E2-R	TCCCCCCGGGATTCTGCGAAGTAATCTGAGT
TEV/CBD-F	CCCGGGCAGATTACGACATTCCAACAACTGATGCAGAGAATTT GTATTTTCAGGGTCGGGCACACCACCACCACCA
HDEL/CBD-R	CTCGAGCTAGAGCTCATCGTGAGAAGTTCCTGATTTTGAGA
18-10F	GGAGAAGAATTTCGATTTGCTTAGTT
18-10R	CTTCGAGACCGTCAA ATCACGGGT
LB-F1	TCATTAATGCAGCTGGCACG
LB-F2	AATTAATGTGAGTTAGCTCACTCATTAGGC
LB-R1	TAGGGTTTCGCTCATGTGTTGAG
LB-R2	GTATTTGTAAAATACTTCTATCAATAAAATTTCTAATTC
RB-F1	GGCTCGCTCGTTTGGAGCTAACAGTACCGTTG
RB-F2	CCTGGATCTGAATGTGTTTGTTTGCAATTTCACGATC
RB-R1	CATGCAAGCTTGGCACTGGCCGTCG
RB-R2	GCAGCTTGAGCTTGGATCAGATTGTCGTTTCC
